# Estimating Mean First Passage Time of Biased Random Walks with Short Relaxation Time on Complex Networks

**DOI:** 10.1371/journal.pone.0093348

**Published:** 2014-04-03

**Authors:** Zhuo Qi Lee, Wen-Jing Hsu, Miao Lin

**Affiliations:** School of Computer Engineering, Nanyang Technological University, Singapore; Hungarian Academy of Sciences, Hungary

## Abstract

Biased random walk has been studied extensively over the past decade especially in the transport and communication networks communities. The mean first passage time (MFPT) of a biased random walk is an important performance indicator in those domains. While the fundamental matrix approach gives precise solution to MFPT, the computation is expensive and the solution lacks interpretability. Other approaches based on the Mean Field Theory relate MFPT to the node degree alone. However, nodes with the same degree may have very different local weight distribution, which may result in vastly different MFPT. We derive an approximate bound to the MFPT of biased random walk with short relaxation time on complex network where the biases are controlled by arbitrarily assigned node weights. We show that the MFPT of a node in this general case is closely related to not only its node degree, but also its local weight distribution. The MFPTs obtained from computer simulations also agree with the new theoretical analysis. Our result enables fast estimation of MFPT, which is useful especially to differentiate between nodes that have very different local node weight distribution even though they share the same node degrees.

## Introduction

Scale-free node degree distribution, small network diameter, large clustering coefficients – these are common properties found to be present in complex networks arising from seemingly disparate fields such as biology, computer science, cosmology, etc. [1], [2]. It is widely believed that there should be common underlying principles behind the formation of these networks that resulted in the observed properties. As such, complex networks have received much research attention during the past decade.

One of the studies pertaining to complex networks is the network efficiency and capacity analysis [3]–[7]. In these studies, answers to the questions such as ‘how fast can a message be delivered to a given destination’ and ‘how many packets may be generated in the system before congestion arises’ are essential in understanding the performance of a network [5], [7], [8]. While there are existing results on routing and flow balancing in networks with certain topologies [9], they usually assumed the underlying topology or the knowledge of link formation mechanism. There are cases where the assumptions do not hold, for example, the animal foraging strategy [10] and the web searching process as depicted by the PageRank algorithm [11]. Approaches based on random walk can be applied when the detailed information of network formation mechanism is absent. Quantities such as *stationary distribution* and *mean first passage time* (MFPT) are important as they can be used to answer the questions about the performance of networks as mentioned above.

The concept of random walk has also been applied in social networks. Even-Dar et al. [12] studied the process of spreading influences in social networks by means of Voter Model and showed that the pathways in which the influences propagate are equivalent to series of random walks. Thus, the MFPT to a node A, yields the expected time for the other nodes in the network to be influenced by node A. Selecting a node with low MFPT for spreading the news could result in fast information propagation.

In fact, MFPT can be calculated by using the fundamental matrix method [13]. However, the computation involves multiple matrix multiplications. When the method is applied on large scale networks with millions of nodes, the computation becomes practically infeasible. Moreover, the solutions obtained from the fundamental matrix approach are too generic and hard to interpret. For instance, it is unclear which factors, be it node degree, eigenvalue, local connectivity, or others, govern the MFPT by just looking at the solution expression. Further research is needed to better characterize MFPT and to reduce the computational cost.

**Figure 1:**
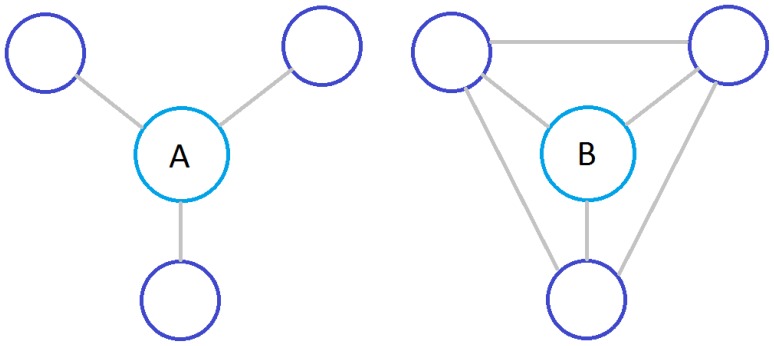
Nodes with same degree may have very different local connectivity. The figure shows two examples where a node with degree 3 may be part of a sparsely-connected star network or a densely-connected clique.

In [10], Condamin et al. showed a mean first passage time analysis using the pseudo Green function. They related MFPT to the network size and diameter. The general applicability of their result to non-fractal networks has been discussed in [14]. Fronczak et. al [8] applied the mean field theory to study the MFPT based on the Erdos-Renyi (ER) random graphs and networks generated by using the Barabasi-Albert (BA) preferential attachment model. Lau et. al [14] showed asymptotic analysis of the first passage time of unbiased random walk for a class of networks with *short relaxation time* by using the mean field theory. However, the solution relates MFPT to the node degree alone. There are cases where the nodes share the same node degree while having vastly different local topology such as those depicted in Figure 1.

Random walks with short relaxation time are also known as random walks with *non-compact exploration* [15]–[17]. In [15], Bénichou et al. presented the conditions for which a random walk on fractal network falls into the compact or non-compact exploration regime. In [16], Hwang et al. presented a fundamental result on the relationship between the node degree and the MFPT of heterogeneous networks, i.e., networks with power-law degree distribution. They focused on uniform random walks without self-loops. It was shown that the general trend of MFPT with respect to the node degree exhibit cross-over behaviour that is governed by the *spectral dimension* 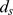 and the exponent of the degree distribution 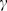. For the case 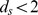, which is known as the compact exploration regime (see also [15]), the MFPT was found to be independent of node degree. For 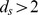, the MFPT is related to the node degree via a power-law function where the exponent is determined by 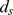 and 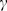.

While the focus of [16] is to show the general trend of MFPT with regard to node degree and spectral dimension, we focus on explaining the differences in MFPT for nodes with same degree when the underlying random walk falls into the non-compact exploration regime. We generalize the FPT analysis as discussed in [14] for a class of random walks with short relaxation time where the nodes have arbitrary weights. In addition, we analyse the first passage time at an improved level of precision by incorporating exact solutions to the stationary distribution. This enables a more detailed view of the neighbourhood around the target node. The new expression allows us to differentiate between the case where the neighbourhood of a node is sparsely connect and the case where it is densely connected as depicted in Figure 1. We will show that the MFPT of a node is closely related to the local weight distribution around it. By changing the node weights, the spectral dimension and hence the relaxation time will be affected, we have also conducted simulations to study how the spectral dimension is affected by varying the weight assignments. They will be discussed in more detail in Discussions section.

## Analysis

### Outline

In this section, we will show the detailed analysis for approximating the FPT decay rate and MFPT of random walks with short relaxation time. First, we will show the stationary distribution for random walk with arbitrary node weight assignment scheme. Then, we apply a flow-based heuristics to estimate the quasi-stationary distribution of the random walk when a sink node is introduced. Next, we show that the FPT distribution follows an exponential decay, and further show that the decay exponent is related to the quasi-stationary distribution of the neighbours of the target node. Finally, we obtain the MFPT by approximating the integration over the FPT distribution.

### Stationary and Quasi-stationary Distribution

A complex network is modelled by a connected and undirected network 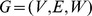, where 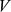 denotes the set of nodes with 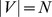, 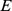 denotes the set of edges 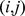, and 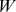 denotes the weights assigned to the nodes. An edge 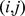 represents the existence of relationship between nodes 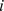 and 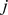. For the sake of simplifying expression, we assume without loss of generality that self-loops are present for all nodes, i.e., 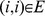 for 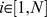. We define a biased random walk on 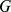 with arbitrary positive weights assigned on nodes. A transition from node 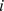 to a neighbouring node 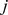 is based on the following transition rule:
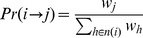
(1)where 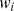 denotes the weight of node 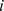, and 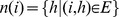 denotes the neighbourhood of node 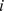. 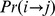 denotes the probability of a random walker at node 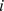 moving to node 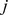 at the next time step. When self-loop is present, the staying probability of a node is not a constant but is dependent on the local weight distribution. Let the transition matrix be denoted by 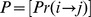, and 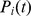 denote the probability of the random walker appearing at node 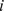 exactly at timestep 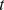. The master equation is given by:



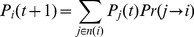
(2)Recall that a Markov Chain (MC) is said to be 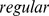 if 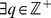, 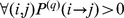 where 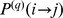 denotes the 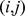 entry of the 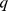-th order transition matrix 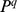. The probability distribution of a regular MC will converge to a unique stationary distribution 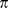 regardless of the starting position as 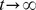. The *relaxation time* 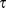 of a Markov Chain is the time for the state probability distribution to be close to the stationary distribution, i.e., the standard deviation of 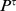 is bounded by 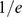 where 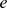 denotes the Euler Number (see [18] Chapter 12). The relaxation time of a Markov Chain is given by 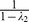, where 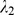 is the second largest eigenvalue [18].

In the networks that we consider, since every node has a self-loop and the graph is strongly connected, the corresponding Markov Chain is regular. In the following, we show the exact form of stationary distribution for the biased random walk as defined in Eq.(1).

Let 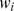 be the weight of node 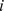, 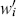 be the neighbourhood weight of node 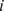 given by 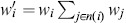, and 
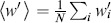
. The stationary distribution of the biased random walk is given by
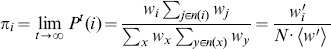
(3)


The expression can be verified by applying the equilibrium condition on the master equation as given by Eq. (2).

Let 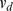 denote the sink node and let 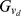 denote the resultant graph from designating node 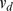 as sink node. Let 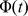 denote the corresponding random walk probability distribution and 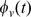 represent the probability for a random walker to be present at node 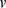 at time 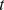. The following set of rules describes the new random walk on 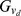:
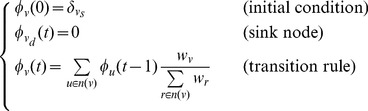
(4)where 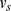 denotes the source node and 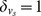 for 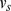, and 0 otherwise. For connected network where 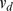 is reachable by every node, 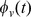 tends to zero when 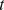 tends to infinity. However, for random walks with short relaxation time [14], [19], the conditional probability distribution 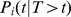 (i.e. conditioned on the survival of the random walker) will converge as 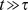, where 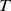 denotes the time to reach the sink node. As such, the converged conditional probability distribution is called the *quasi-stationary distribution* denoted as 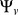. For 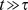, we have the following approximation:
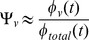
(5)where 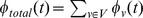 denotes the total survival probability at time step 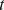.

We approximate the quasi-stationary distribution of 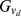 around the sink node with a flow-based heuristic [14] described in the following. We begin with the stationary distribution 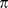 in 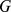. Under the stationary distribution, based on Eq.(3), the probability of the random walker traversing an edge 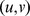 is 
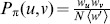
 Next, we treat an undirected edge as a combination of in-link and out-link. We remove the out-links from 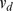 and hence making 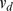 a sink node. Thus, the equilibrium will be broken and nodes 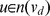 will have their ‘flow’ constantly drawn by 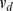. Finally, we approximate the quasi-stationary distribution of such nodes by discounting the probability of utilizing the edge 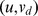:
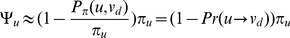
(6)


### Asymptotic First Passage Time Analysis

To obtain FPT, we re-designate the destination node 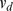 as a sink node such that the random walk process terminates once the random walker moves into the sink node. The time taken for it to be absorbed into the sink node is then the same as the FPT. Let 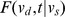 denote the probability of visiting node 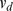 at timestep 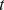 for the first time with 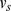 as the starting node. Then by definition, we have
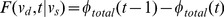
(7)i.e. the first passage probability at time 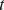 is given by the difference in the total survival probability between time 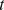 and 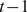.

On the other hand, we can also obtain the first passage probability by using the transition rule.
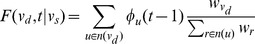
(8)


For 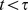, the first passage probability is dependent on the source-sink distance. Here, we focus on the asymptotic behaviour of the random walker for 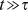. As such, we can apply Approx.(5) and after simplifying, we get:
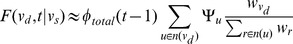
(9)


Let 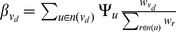. By combining Eq.(7) and Approx.(9), after simplification, we obtain the following recursive approximation:
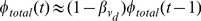
(10)


By definition, 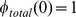 as the random walker has not started moving and thus the survival probability is 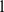. Solving the recursive formula yields:
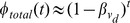
(11)

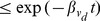
(12)


Substituting the result back to Approx.(9) yields:
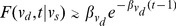
(13)


Thus, the first passage probability is approximately bounded below by an exponential function for 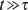 and the decay rate is given by 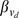. To calculate the decay rate, we apply Approx.(6):
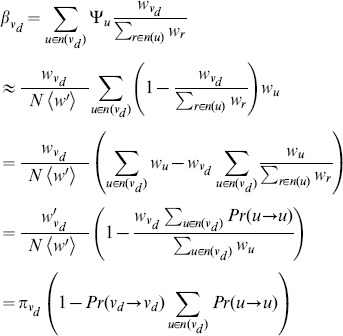
(14)where the last step is just a simplification of the expression by using the definition as shown in Eq.(1) as we assumed earlier that every node in the network has a self-loop. Nevertheless, for the cases where self-loop is absent, the expression 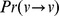 can be substituted for 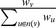, as self-loop played no role in our derivation except for the regularity of the random walk.

The result in Approx.(14) suggests that the decay rate of the first passage time distribution depends mainly on the sink node’s stationary distribution and the transition probabilities around the sink node.

### Mean First Passage Time

**Figure 2:**
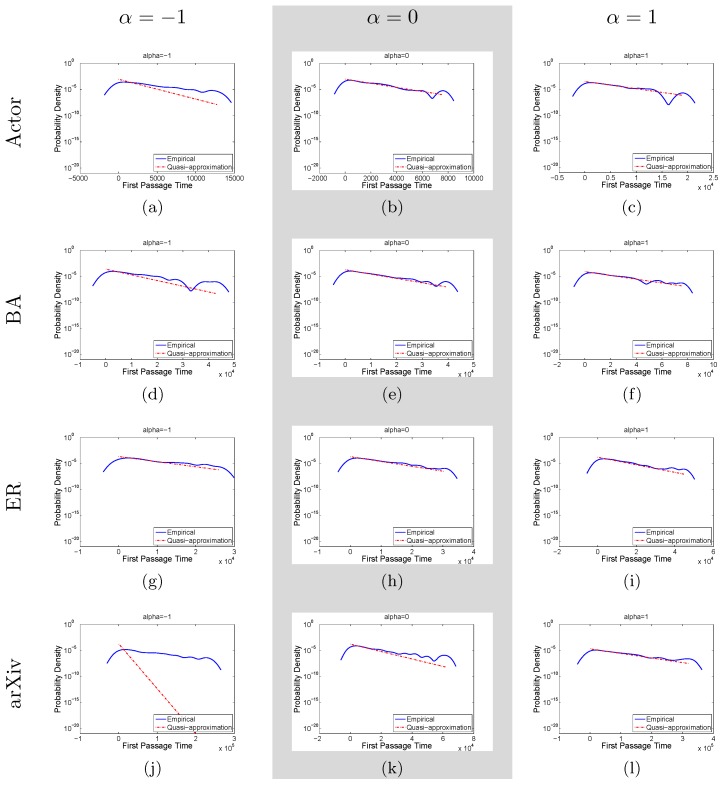
Plots of empirical first passage time distribution against theoretical prediction according to the approximate bound given by Ineq.(13) for different networks and weighting factors. Each row corresponds to a network in the following order: Actor, BA, ER, and arXiv. The columns, from left to right, correspond to 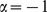, 0, 1 respectively. For most cases, the tail of the first passage time distribution can be predicted fairly accurately except for Figure 2(j), which is due to the high relaxation time as shown in Table 2.



After obtaining the first passage time distribution, we can estimate the mean first passage time by using the approximation:
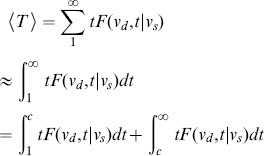
(15)for some 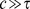. In Approx.(15), the integral is separated into two parts – from 1 to 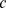 and from 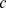 to 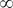 for short-term and long-term behaviour respectively. While the first passage time analysis is based on the assumption 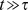, our result is an upper bound on the first passage probability for small 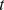. This is justified by the observation that the first passage time probability starts from zero initially, increases to a peak, then decreases exponentially (see Figure 2 and [15] for non-compact case) with the decay rate as presented previously. Thus, Ineq.(13) is an overestimation of the first passage probability for small 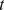. Hence, we provide a lower bound of mean first passage time as follows:

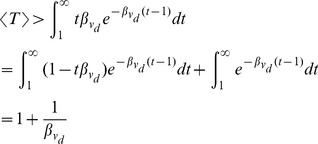
(16)Although Ineq.(16) is a lower bound of the mean first passage time, results from simulations show that it is strongly correlated to the actual MFPT.

In order to calculate the MFPT, an edge will be followed exactly twice to obtain the weight of the connected neighbour. Thus, assuming the network is represented in adjacency list format, the time complexity of the approximation is given by 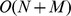. The time complexity to convert a network from adjacency matrix representation into adjacency list representation is bounded by 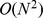. Hence, the overall time complexity of the approach is bounded by 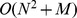 for adjacency matrix representation. Nevertheless, in terms of computational efficiency, the approach is a great improvement from the fundamental matrix method [13] which has time complexity of 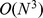 or 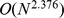 depending on the actual implementation of matrix operations [20]. Thus, our approach can be used to estimate MFPTs of the network quickly especially for large scale networks.

## Results

We verify the theoretical results on two real world networks and two artificially generated networks: (i)arXiv General Relativity and Quantum Cosmology collaboration network, obtained from the Stanford SNAP website [21], (ii) Barabási-Albert (BA) preferential attachment network [1], [22], (iii) Erdos-Renyi (ER) random graph, and (iv) Actor collaboration network from the Barabási lab [1]. Because the analysis only applies to connected graphs, we used the largest component of the arXiv network. The two generated networks are chosen to test the theoretical results on networks with different structure and edge density. The ER network is generated with 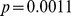, while the BA network is generated with 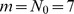. The Actor network is constructed from the first 1000 records of the database, where each record consists of the actors who collaborated in the same movie. Table 1 summarizes the networks that we have examined.

**Table 1:** Summary of networks studied.

Network	# nodes	# edges	CC	Dm	Source
arXiv	4158	15501	0.5569	17	[19]
BA	4158	31136	0.0174	5	[1]
ER	4158	21020	0.0022	7	[13]
Actor	968	13324	0.6751	9	[1]

C denotes the average clustering coefficients and Dm denotes the network diameter.

We apply the node weight assignment scheme given by 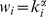, where 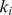 is degree of node 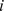 and 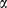 is an integer. This node weight assignment scheme is mainly studied in the network traffic community such as [5], [7]. For our experiments, we mostly consider the range of 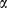 in 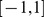 except for the Actor network as other values with greater magnitude will result in random walks with exceedingly long walk lengths.

The experiments are conducted as described below. Firstly, for a given network, we choose 20 nodes randomly as the source nodes. The weighting factor 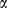 is then fixed and the node weights are computed accordingly. For each source node, 250 times of full random walk simulations are conducted independently. A full random walk simulation starts with the random walker at the source node and terminates when every other node has been visited at least once. The first passage time to each node is recorded. The procedure is then repeated for other values of 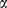.

To obtain the first passage time distribution, we applied Gaussian Kernel Density Estimation on the first passage time statistics collected from the simulations. We randomly selected different source-sink pairs for each network and different values of 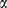, and the results are plotted in Figure 2. As shown in the figure, for most cases, we can predict the tail of the first passage time distribution fairly accurately except for the case when 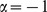 in arXiv network. With reference to Table 2, we find that for 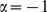, the relaxation time for the corresponding random walk is very high, and thus for certain sink nodes with high absorption rates, our theoretical result may not be applicable.

**Table 2:** Pearson’s correlation coefficient for MFPT.

Network	Correlation			Relaxation time *τ*		
	*α = *−1	0	1	*α = *−1	0	1
arXiv	0.2619	0.7435	**0.9269**	555.56	555.56	1666.7
BA	**0.9241**	**0.9812**	**0.9940**	3.5311	2.2060	2.0178
ER	0.4860	**0.9846**	**0.9973**	16.502	3.7327	2.8417
Actor	0.0064	**0.9231**	**0.9851**	476.19	61.728	26.041

Overall, the correlations are high whenever the relaxation time is low. For the BA network, the relaxation time is consistently low and thus the correlation is extremely good.

**Figure 3:**
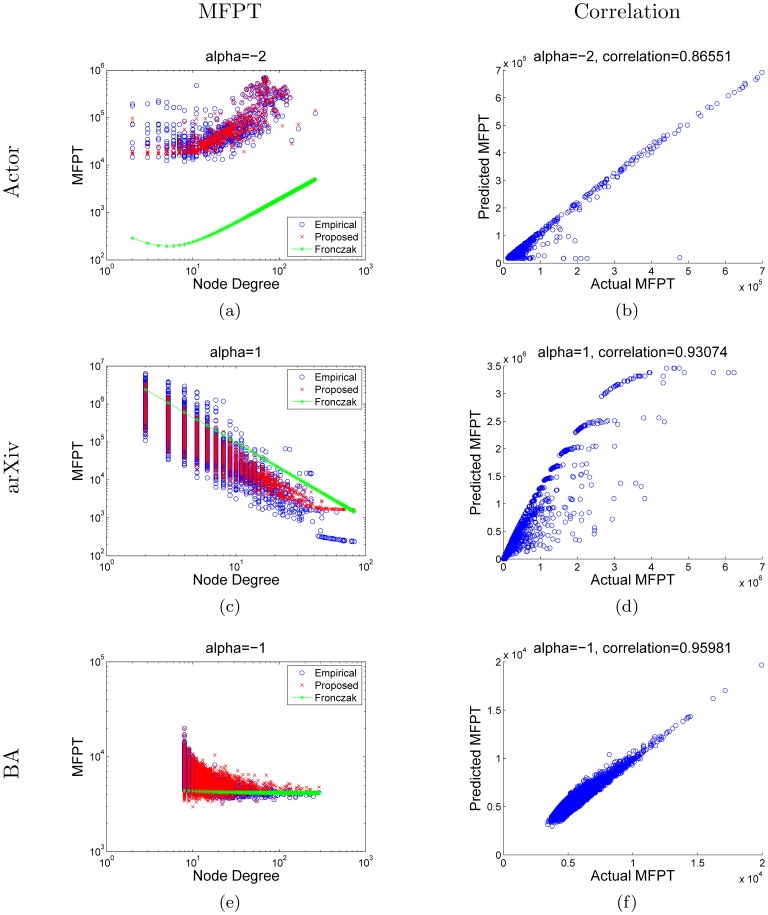
Comparison of MFPTs obtained from simulation against that obtained from using the bound shown in Ineq. (16). The column on the left compares the empirical MFPT to the results obtained by (i) using the bound in Ineq. (16); (ii) using the result presented in [8]. The column on the right shows the scatter plot and correlation between the empirical results and our proposed theoretical results. The rows correspond to the Actor, arXiv, and BA network respectively. While the result presented by Fronczak et al. [8] gives the general trend of MFPTs with respect to node degree, we find that the MFPTs for a given node degree are distributed across a wide range and cannot be fitted with a function of the node degree alone. The scatter plots show a strong correspondence between the empirical results and our proposed theoretical results. This is further supported by the high Pearson correlation coefficients which are shown on top of the scatter plots.

In Figure 3, we compare the empirical MFPT to that predicted by Ineq. (16). Since we relaxed several expressions during the derivation, the values predicted by Ineq. (16) may not be of the same scale as the empirical result. Therefore, we renormalized the predicted values *(P-set)* with respect to the empirical result *(E-set)*. The renormalization scheme is described as follows. First, we sort both the P-set and E-set in non-increasing order. Then we rescale the middle 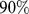 of the P-set with respect to that of the E-set, i.e. obtain the rescale parameters (shearing and scaling) by ignoring both the upper and lower 5 percentile of both sets. Finally, the renormalization is applied to the whole P-set.

As shown in Figure 3, we can observe that while the result by Fronczak et al. predicted the general trend of MFPT with respect to node degree, our result further refined the predicted values by examining local weight distribution. By zooming into a greater level of detail, our approach has revealed a useful relationship between local connectivity and the MFPT especially as highlighted in Figure 3(a),(c), and (e). The weighting factor 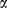 controls whether high degree nodes should receive greater attention or vice-versa. Thus, for small 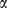, high degree nodes should be reached less often and hence greater MFPT. Surprisingly, even for 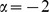, there are cases where high degree nodes can be reached fairly quickly as depicted in Figure 3(a). By examining the structure of the Actor network, we find that many high degree nodes are connected with bridge nodes of low degree. On the other hand, the MFPT of the BA network does not show this kind of pattern. We believe that this is due to the fact that the BA network possesses a much simpler structure than the Actor network.

To investigate the overall performance of the bound given in Ineq. (16), we also calculated Pearson’s correlation coefficient, and the results are summarized in Table 2. Overall, the correlations are high whenever the relaxation time is low. For the BA network, the relaxation time is consistently low and thus the correlation is extremely good.

## Discussions

**Figure 4:**
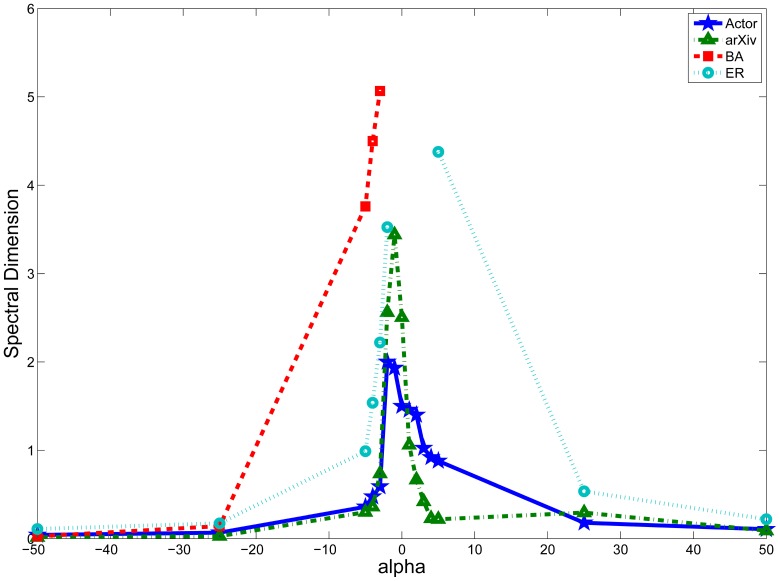
Relationship between spectral dimension 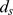 and the weighting factor 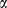. The data used for drawing this figure is tabulated in Table 3. The lines corresponding to BA and ER network appear disconnected as they have 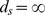 for certain values of 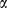 and cannot be adequately shown in the figure. The spectral dimension generally peaks in the interval [−1, 1] and drops significantly for 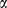 of greater magnitude. This is especially true for the BA and ER networks (from infinity to a finite value). In the extreme case, by setting 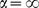, the ‘random walk’ is no longer random as the node with largest degree will always be chosen at every step. Similar reasoning also applies for 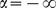. Therefore, towards both extremes, we would expect the random walk to become more localized and hence falls into the compact exploration regime.

In [15], [16], it was found that the random walk exploration can be divided into compact and non-compact regimes based on their spectral dimension. For the compact case, the random walker spends longer time travelling around certain neighbourhood and hence the relaxation time is long, while the non-compact case is the other way round. In our study, we focus on the non-compact case, i.e., random walk with short relaxation time. While the structure plays a part in deciding the dynamics of random walk, the weighting factor 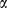 also affects the mixing rates. To examine how much the assumption of short relaxation time holds when the weighting factor 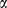 is changed, we also investigated the relationship between 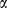 and the spectral dimension of the corresponding random walk 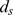. We numerically estimate 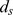 by using the following relation [17]:
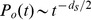
(17)where 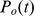 denotes the Return-to-origin (RTO) probability at time 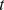 [17]. To obtain the RTO probabilities, we conducted simulations with 100,000 random walkers which are placed randomly at 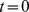. Each random walker walks independently for 100,000 time steps, and the fraction of random walker returning to their respective starting node is recorded at each time step. Table 3 and Figure 4 summarizes the result.

**Table 3:** Spectral dimension *d_s_* w.r.t. weighting factor *α*.

		*d_s_*		
*α*	Actor	arXiv	BA	ER
−50	0.042	0.016	0.026	0.110
−25	0.068	0.028	0.142	0.173
−5	0.360	0.300	3.760	0.990
−4	0.476	0.360	4.500	1.537
−3	0.590	1.735	5.066	2.220
−2	2.000	2.561	∞	3.526
−1	1.932	3.440	∞	∞
0	1.500	2.505	∞	∞
1	1.456	1.060	∞	∞
2	1.400	0.667	∞	∞
3	1.025	0.420	∞	∞
4	0.920	0.229	∞	∞
5	0.880	0.220	∞	4.378
25	0.180	0.295	∞	0.536
−50	0.107	0.090	∞	0.220

The data is obtained by conducting simulation and fitting the exponent of RTO probability according to the definition given in Eq.(17). We mainly consider the range [−5,5] as they are mostly considered in the literature. The entries {−50, −25, 25, 50} are used to examine the effect on 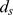 for large 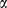. The entries with 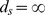 are obtained by the observation that the RTO probability stabilized fairly quickly in less than 10 time steps. The data in this table is plotted in Figure 4.

As shown in Figure 4, the spectral dimension generally peaks in the interval [−1, 1], which explains the applicability of our result. While for the case of uniform random walk, it was shown that 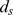 of both BA networks and random graphs are infinity [23], we found finite 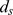 for 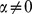. In the extreme case, by setting 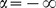, the ‘random walk’ is no longer random as the node with smallest degree will always be chosen at every step. For this scenario, the network will be broken into cycles where leaf nodes form the smallest cycle. Similar phenomenon also applies for 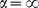. Therefore, towards both extremes, we would expect the random walk to become more localized and hence falls into the compact exploration regime where our result may not be applicable.

**Figure 5:**
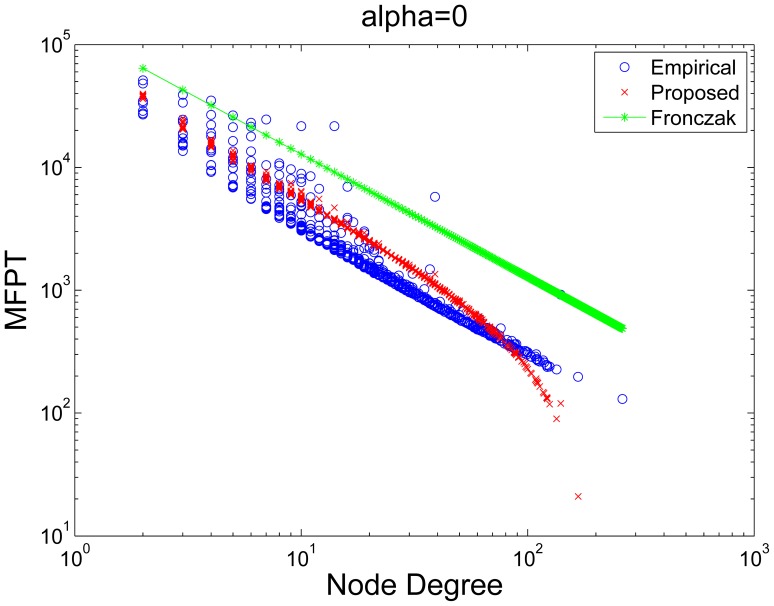
MFPT of the Actor network when 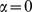. Even though the spectral dimension 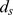 is less than 2, the MFPT is not found to be independent of node degree. Instead, the MFPT exhibits a power-law relationship with respect to the node degree. The disparity arises probably as a result of self-loops, which affects both the RTO probability distribution and the estimated 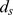.

We also observe several disparities between our results and that of [16]. For instance, as shown in Figure 5 and Table 3, when 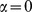, we found that the spectral dimension of the Actor network is 1.500. However, the MFPT is found to be following a power-law relationship with respect to node degree instead of being independent of node degree. Similar results have been obtained for the arXiv network for 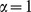, for which 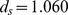, as shown in Figure 3(c). We believe that the disparities arise from the following facts: (i) we considered random walks with self-loops where the staying probabilities are proportional to the node weights; and (ii) the random walks are biased by weight assignments. The self-loop changes the RTO probability distribution and thus also affects the estimated 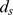. The nodes are weighted differently, therefore the cross-over threshold for 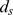 may not be the same as that of [16]. Further research is needed to better understand the effects of self-loops and node weights on the spectral dimension of a random walk.

In summary, we have shown the exact form of stationary distribution for a class of biased random walks on networks where the nodes are assigned arbitrary weights. By using this result, we have presented a new method that gives improved estimation of MFPT for random walks with short relaxation time. We have verified that the decay rate of the first passage time distribution can be estimated fairly accurately and the MFPTs are found to be better revealed by local weight distributions. Given its low computational cost, our method enables quick inspection of the MFPT for large scale networks. This is especially true for cases where the ranking rather than the actual values of MFPT of nodes is more important. For instance, to contain virus outbreaks, the new method can be used to quickly rank the nodes based on estimated MFPT and judiciously apply security measures on the nodes that are ranked highly. Our result can also be readily extended to the case of cyclic search [8] where the random walker will scan the direct neighbours of current node for the target as opposed to blindly following the transition rule.
